# Impact of Basalt Filler and Ceramizable Additives on the Toxicity of Gaseous Products Emitted from Thermal Decomposition of Silicone Rubber Composites

**DOI:** 10.3390/ma12213478

**Published:** 2019-10-24

**Authors:** Przemysław Rybiński, Bartłomiej Syrek, Witold Żukowski, Dariusz Bradło

**Affiliations:** 1 Institute of Chemistry, The Jan Kochanowski University, Żeromskiego 5, 25-369 Kielce, Poland; bartlomiejsyrek@wp.pl; 2 Department of Chemical Engineering and Technology, Cracow University of Technology, Warszawska 24, 31-155 Kraków, Polanddariusz.bradlo@gmail.com (D.B.)

**Keywords:** basalt flake, basalt fibres, silicone rubber, toxicity, fire hazard

## Abstract

The article illustrates the impact of basalt filler in the form of flakes and fibres on the toxicity of gaseous products that formed during the thermal decomposition of silicone rubber composites. The values of specific emissions of gases were determined with the help of the IR spectroscopy and further applied to calculate the toxicometric index. The presented method of measuring the concentrations of gaseous products resulting from thermal decomposition consists in the application of a fluidised bed reactor, which makes it possible to conduct the decomposition of a sample at a precisely assumed temperature value and imitate the conditions of a real fire. At a temperature lower than 700 °C, the gases resulting from the thermal decomposition of composites are particularly toxic due to the presence of significant concentrations of formaldehyde that does not undergo oxidation to more stable inorganic products. At a temperature of 600 °C the toxicity of gases for the samples with ceramizable additives and without them was similar. In the first case, there appeared to be a positive synergistic effect of mineral and basalt additives, and the basalt additives themselves increased the toxicity of gases. At higher temperatures of decomposition, the exponentially increasing rate of the oxidation reaction in the gaseous phase results in the lack of significant differences between the toxicity of gases for the samples with and without basalt additives. The toxicometric index value at temperatures of 700 °C and 800 °C was by one or two orders of magnitude higher, respectively, than the one that was observed in the temperature range of 500–600 °C, as inorganic components appear in the place of formaldehyde.

## 1. Introduction

Polymers and their composites are commonly used in almost all spheres of life. The manufacture and global consumption of polymers and materials produced from them has been steadily increasing in spite of numerous ecological restrictions mainly related to the limitation of producing considerable amounts of production and post-consumer wastes, which are frequently difficult to manage. The problem of reducing their flammability or flame retardancy is becoming particularly important due to a serious health and life threatening aspect, as well as the pollution of natural environment that is caused by the emission of smoke and toxic gaseous products of their thermal decomposition and combustion. Having analysed the literature on the subject, one might draw a conclusion that 55–80% of all fatal accidents during fires are caused by poisoning with products of thermal decomposition and combustion as well as smoke [1,2,3]. Toxic gases easily enter the human body as a result of inhalation and absorption through the skin.

During the thermal decomposition and combustion of polymer materials, the following toxic compounds are most frequently formed: carbon monoxide, hydrogen chloride, hydrogen cyanide, nitrogen and sulphur oxides, nitro compounds, and organochlorine derivatives, including polychlorinated dibenzo-p-dioxins and dibenzofurans [4,5]. The emission of polycyclic aromatic hydrocarbons (PAHs) also poses a serious threat [6,7]. The first six substances that are mentioned above are among the most harmful ones that determine the toxicity of the environment. This is due to the fact that they are released in the biggest amounts and their impact on the human body is quick, whereas the values TLV®-TWA—Threshold Limit Value—8-hour Time Weighted Average are very low [8].

The basis for the activities aimed at reducing the poisoning hazard are the laboratory research results, including the measurements of the following: the potential rate of the formation of thermal decomposition and combustion products, the amount and rate of smoke emission under fixed combustion conditions, the emitted amount of heat, an increase in oxygen deficiency, and the mutual relationships of these magnitudes. When analysing the polymer materials under the same kinetic and thermal conditions, one might estimate their behaviour in fire conditions. Such tests also make it possible to make attempts to modify the composition of these materials in order to e.g. improve fire retardancy or reduce the emissions of toxic products during combustion.

Currently, the assessment of the toxic potential of a gas mixture resulting from a fire is based on the Fractional Effective Dose (FED). The necessary condition for the most reliable determination of the FED value is a precise qualitative and quantitative analysis of toxic products that are generated during a fire [9,10]. The most popular, a coupled technique of thermogravimetric analysis/Fourier transform infrared spectrometry (TG/FTIR), cannot give detailed information regarding gas composition and, thus, some additional tools are used. Examples can be pyrolysis gas chromatography/mass spectrometry (Py-GC/MS) [11,12] or mass spectrometry (TG-FTIR-MS) [13], which can both significantly improve qualitative analysis with general insight into the quantity of measured gases or concentration of its components. An interesting, but still not fully-qualitative, approach was proposed by a Chinese team [14,15]. Their semiquantitative FTIR method was based on the principle that the relative yields of functional groups and gaseous products could be assessed by the integral of absorbance curves. The method of measuring concentrations of toxic thermal decomposition products that was proposed in this article is based on using a fluidised bed reactor and the infrared spectrum analysis making use of the Michelson interferometer and Fourier transformation (FB-FTIR). This allows for the reproduction of the conditions corresponding to the immediate effect of a high temperature on the sample, like in the case of a real fire. The fluidised bed also ensures adequate heat and mass exchange, which causes that the whole process to take place intensely and it is characterized by a high rate and a relatively short time of decomposition. Undoubtedly, the great advantage of the proposed method, in relation to the laboratory methods currently used, is the possibility of a simultaneous qualitative and quantitative analysis of released toxic gases. The determination can be carried out at a strictly defined temperature, including that corresponding to the maximum rate of decomposition of the tested material, which directly translates into the accuracy of determining the FED dose or toxicometric index [16]. The FED approach to determine toxicity of gases emitted form materials in the case of fire is generally accepted and commonly used [17,18]. Nonetheless, in the present work, a more universal solution for evaluation of relative toxicity of complex, multicomponent gas mixtures was proposed.

The article presents research results referring to the impact of basalt filler in the form of both flakes (BFL) and fibres (BFS) on the toxicity of gaseous products of the thermal decomposition of silicone rubber composites, including the composites containing ceramizable additives. Basalt fibres were proven to be an environmentally friendly and promising additive to polymer, metallic, and concrete composites [19], however its impact on the toxicity has not been presented yet. The problem of the emission of toxic gases during their decomposition is of key importance from the point of view of fire safety [20,21,22,23,24,25,26] if we take into consideration that ceramizable composites are at present more and more commonly used as an insulation material for wires that are intended for supplying power to electrical devices in huge malls, on the boards of planes, or in lifts. An earlier article presents the effect of basalt fillers on the thermal properties and flammability of silicone rubber composites, including ceramizable composites [27].

## 2. Materials and Methods

### 2.1. Materials

The polymer matrix was methyl vinyl silicone rubber (SR), which was made by Silikony Polskie (Nowa Sarzyna, Poland), with a vinyl group content of 0.05–0.09% (mol × mole^−1^). The rubber was cross-linked with the use of dicumyl peroxide from Sigma Aldrich (Schnelldorf, Germany) in a quantity of 2.5 parts by wt./100 parts by wt. of the rubber. 

The following compounds were used as fillers in the rubber blends: Aerosil 200 fumed silica (BET specific surface area 175–225 m^2^/g; mass loss on drying ≤1.5%, obtained from Evonik Industries (Wesseling, Germany)); melamine cyanurate (MC) (trade name Evermel Active 47, obtained from Everkem, Milano, Italy); and, calcium carbonate anhydrous, free-flowing, reagent ≥99% (obtained from Sigma-Aldrich, Schnelldorf, Germany). The ceramization-promoting glass frit “A 2120” of the chemical composition (wt.%) 7.3CaO-25Na_2_O-2.1K_2_O-4.2Al_2_O_3_-61.4SiO_2_ and softening point temperature of 540 °C was obtained from Reimbold & Strick GmbH, Cologne, Germany; zinc borate ZnO ≥ 45%, B2O ≥ 36% was obtained from Sigma-Aldrich (Essen, Germany); basalt flakes (BLF) and basalt fibres (BFS) (trade name BCS13-6.35-DRY length 0.02 m; width 0.02 m; height 0.022 m, obtained from Basaltex (Wevelgem, Belgium)).

### 2.2. Methods 

The composite mixes (Table 1) were prepared while using a laboratory two-roll mill (the rolls had the length of 200 mm, the diameter of 150 mm, and they were sourced from Bridge City, UK), working with a rotation speed of 18 rpm (revolutions per minute) for the slower roll and 20 rpm for the faster roll (friction 1:1). The kinetics of vulcanization of the composite mixes was tested by means of a rheometer (Alpha Technologies MDR2000 (Alpha Technologies, Hudson, OH, USA)) according to the ISO 37:1994 standard (temperature: 160 °C, frequency: 1.667 Hz, strain: 0.5°). According to the results, the samples were shaped and then vulcanized in steel moulds by a laboratory press at 160 °C under 10 MPa of pressure.

The carbon content in the tested composites (%C) is the weighted average of the carbon content in the individual components of the composite. The balance of this element was also estimated based on the theoretical carbon content in the tested samples, giving the ratio of the mass of carbon atoms in the flue gas, in relation to the initial material subjected to combustion.

**Figure 1:**
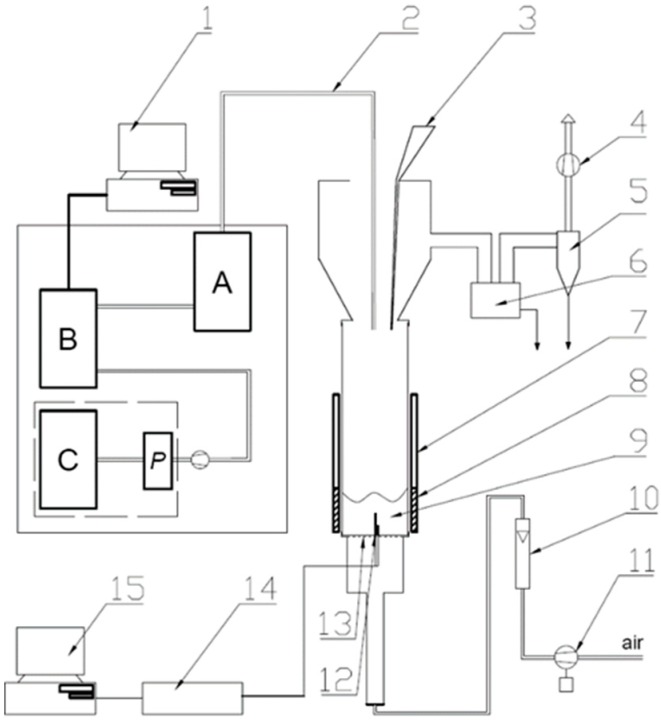
Scheme of fluidised bed reactor: 1—computer storing data from FTIR analyser; 2—heated probes for sampling flue gases; 3—batcher; 4—exhaust fan; 5—cyclone; 6—ash trap for coarser particles; 7—movable radiation shield; 8—heating jacket; 9—bubbling bed; 10—air rotameter; 11—blower, for fluidising air; 12—two thermocouples; 13—flat, perforated metal plate distributor; 14—A/D converter for thermocouple signals; 15—computer storing chemical analyses data and temperature; A—mobile conditioning system of Gasmet DX-4000, B—FTIR analyser (Gasmet DX-4000), C—Horiba PG250, P—Peltier cooler.

The gaseous products that were generated during the thermal decomposition of silicone rubber composites have been analysed in terms of quantity and quality. A laboratory set (Figure 1) using fluidised bed technology was used for this purpose. The essential part of the set was a fluidised bed reactor in the form of a quartz tube with an outer diameter of 100 mm, a length of 500 mm, and a wall thickness of approx. 2 mm. The reactor was placed on a perforated plate (distributor) that was made of Cr-Ni alloy with a thickness of approx. 1 mm. Fluidising gas was air, which provided oxidising conditions in the reaction system. The aluminosilicate microspheres (300–355 μm diam., 200 g) were used as a fluidised bed. The air flow was maintained at about 25 dm^3^/min. while using a TSI 40241 flow meter. The gas composition analysis was carried out at four temperatures: 500 °C, 600 °C, 700 °C, and 800 °C, corresponding to the decomposition temperature of the tested composites. A resistance heating mantle that was connected to the autotransformer was used in order to heat the bed and stabilize the temperature. The Ni-NiCr thermocouple placed inside the reactor, 50 mm from the distributor, was used to measure the temperature of the bed. The masses of silicone rubber samples and its composites have been experimentally selected so as not to exceed the absorbance value equal to 1 in the range characteristic of the silicone rubber matrix (800–1300 cm^−1^). A composite sample in the form of solid pieces of appropriate mass (from 0.030–0.054 g) was placed at the top of the reactor and it was dropped to the bed where, due to the densities difference, the sample was immersed and eventually landed at the bottom of the reactor. At a given temperature of the bed, the samples were dosed in sequence at regular intervals (lasting from 2 to 20 min.), while three samples were tested for each type of composite. The concentrations of gases during thermal decomposition were continuously analysed and, after the concentrations of the main components (CO_2_, H_2_O, and CO), reached the background level, the next sample was placed in the reactor. The presented results are the arithmetic means obtained from three samples of a given type of composite.

Quantitative and qualitative data were obtained while using the Gasmet DX-4000 analyser. The device that is equipped with the Michelson interferometer and a gas cuvette with an optical path length of 5 m allows for obtaining the e-m spectrum of exhaust gases while using infrared spectroscopy with Fourier transform (FTIR). The FTIR spectra of gases were continually collected every 5 s during the experiment. Using the library of reference spectra, the computer program deconvoluted the obtained spectra and, in this way, the concentrations of the following organic and inorganic compounds were determined: H_2_O, CO_2_, CO, NO, NO_2_, N_2_O, NH_3_, CH_4_, C_2_H_6_, C_3_H_8_, C_2_H_4_, C_6_H_14_, HCHO, CH_3_CHO, HCN, and cyclic siloxanes: D_3_ (hexamethylcyclotrisiloxane), D_4_ (octamethylcyclotetrasiloxane), and D_5_ (decamethylcyclopentasiloxane). In addition, the Horiba PG250 analyser was used to verify the measurements while using other detectors: for measurement of the CO, CO_2_, and SO_2_ concentrations the NDIR detectors were used, the NO and NO_2_ concentrations were measured as the sum of NO_x_ by the chemiluminescence method, and the O_2_ volume fraction was measured by the electrochemical detector.

## 3. Results and Discussion

### 3.1. FTIR Spectra Analysis

**Figure 2:**
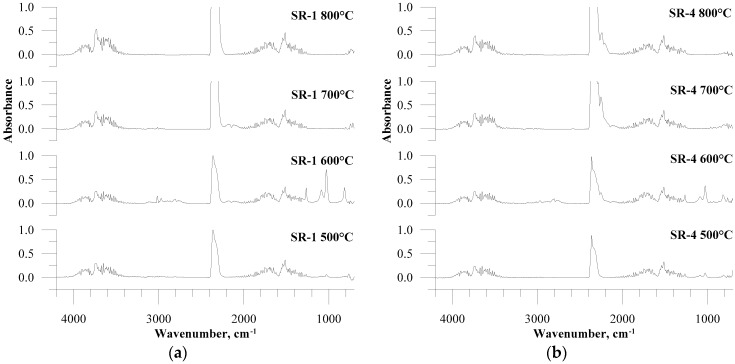
Fourier transform (FTIR) spectra of gaseous products of silicone rubber composites thermal decomposition: (**a**) SR-1; (**b**) SR-4 at examined temperatures.

The FTIR spectra of the gaseous product emitted during the reference cross-linked silicone rubber (SR-1) decomposition in the fluidised bed showed the presence of signals originating from CO_2_ (2250–2400 cm^−1^), H_2_O (3500–4000 cm^−1^; 1300–1900 cm^−1^), and low molecular organic compounds, primarily aldehydes and aliphatic hydrocarbons (1300–1500 cm^−1^, 2600–3200 cm^−1^) (Figure 2a). It is also clearly visible that, at a low temperature, below 700 °C and especially at 600 °C, a signal appears in the spectral range of 800–1300 cm^−1^ in the form of four bands corresponding to the presence of cyclic siloxanes. The FTIR gas spectra of ceramizable composite reference sample (SR-4) thermal decomposition products (Figure 2b) do not differ significantly from the spectra of SR-1. The main distinction is the presence of additional bands in the range of around 2000-2300 cm^−1^ at a temperatures over 600 °C. This is due to melamine cyanurate (MC) introduction (in an amount of 30 phr) to ceramizable composites. MC gradually decomposes, first producing ammonia, which is then oxidised to nitrogen oxides, of which N_2_O (with analytical area of 2000–2222 cm^−1^) is particularly characteristic. The other difference between the SR-1 and SR-4 spectra can be lowering the absorbance in the range of typical cyclic siloxanes when significant amounts of fillers are introduced into the polymer matrix in the case of ceramizable composites. The introduction of basalt flakes or basalt fibres to the composites did not substantially alter the spectra, thus these spectra were not shown. Nevertheless, the computer software performed deconvolution of each spectrum obtained during the experiments and enabled one to qualitatively compare the influence of these fillers on the emission and the toxicity of silicone rubber composites.

### 3.2. Concentrations of Emitted Gaseous Products

**Figure 3:**
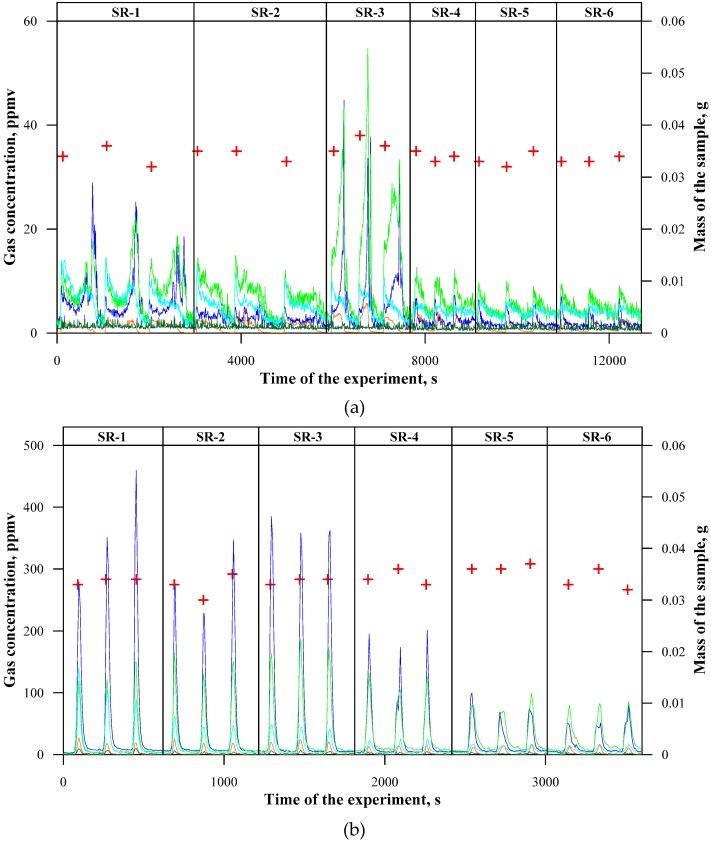

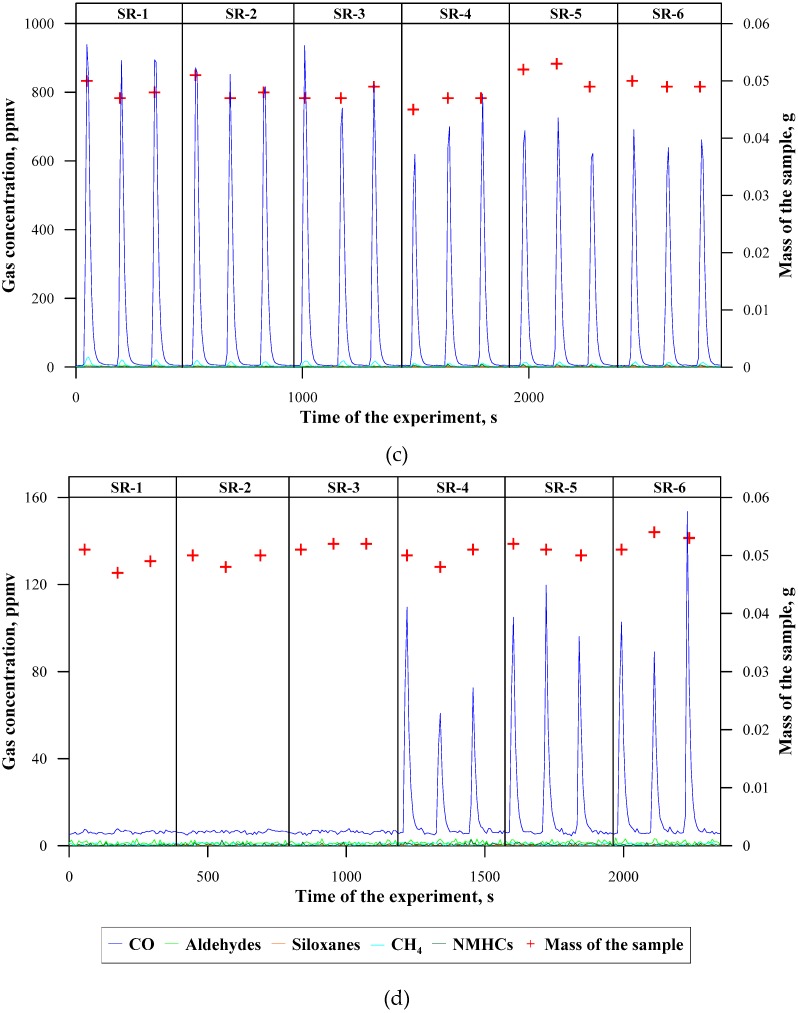
Concentrations of the main toxic products emitted during thermal decomposition of silicon rubber composites: (**a**) at 500 °C; (**b**) at 600 °C; (**c**) at 700 °C; and, (**d**) at 800 °C.

**Figure 4:**
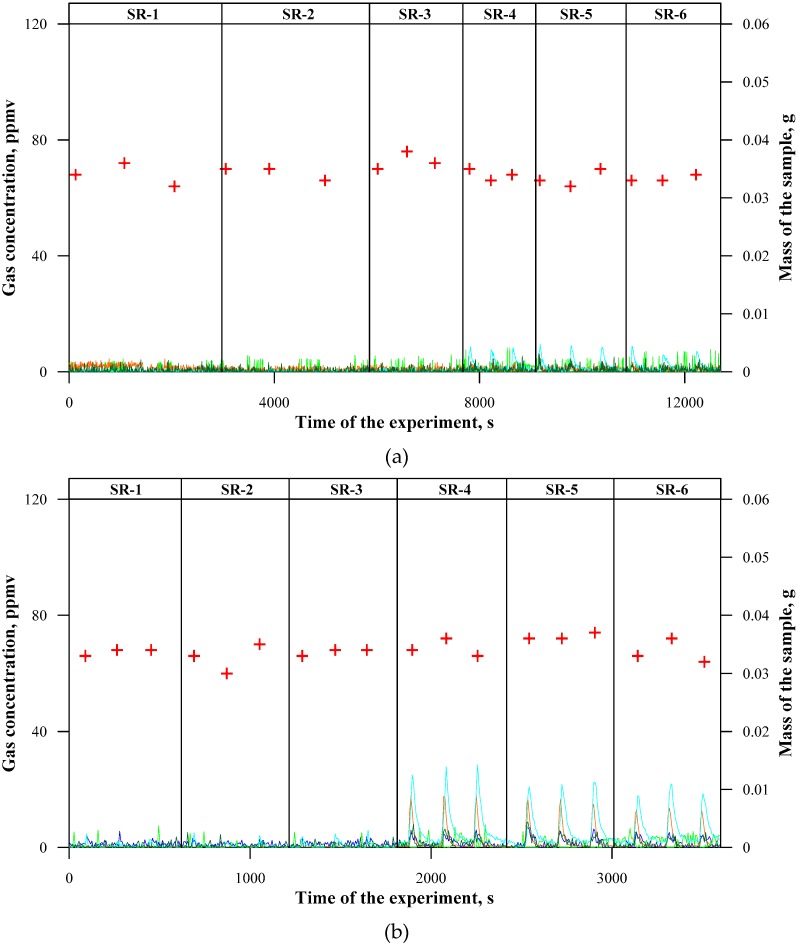

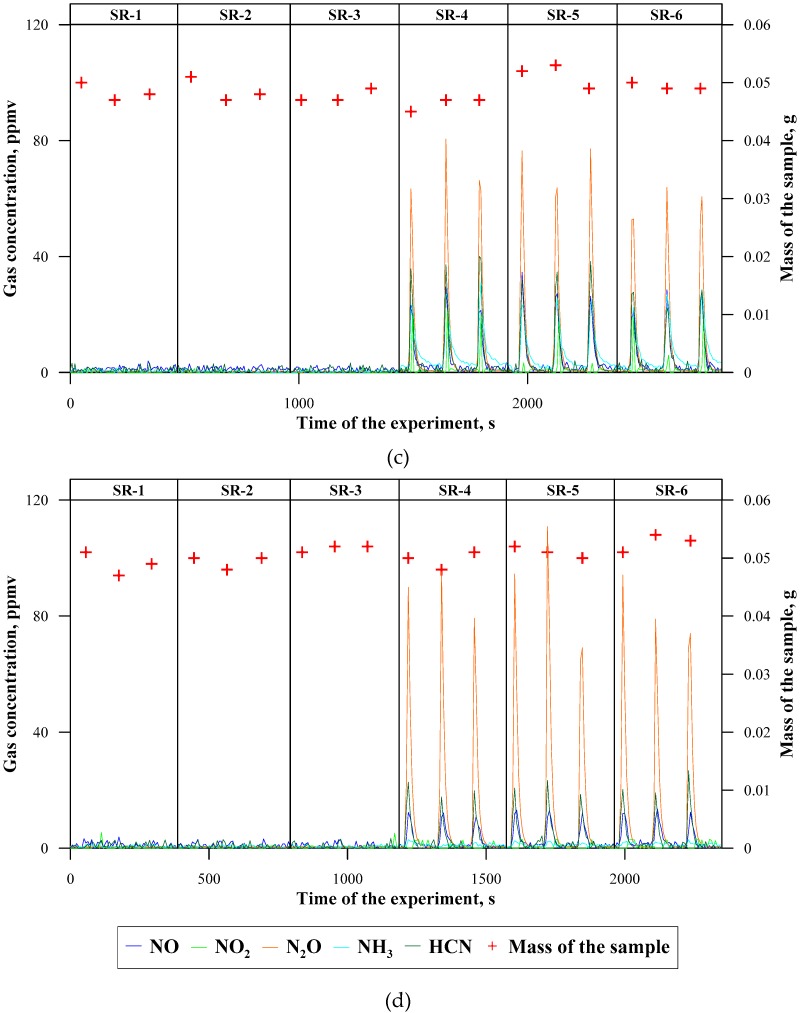
Concentrations of nitrogen compounds emitted during thermal decomposition of silicon rubber composites: (**a**) at 500 °C; (**b**) at 600 °C; (**c**) at 700 °C; and, (**d**) at 800 °C.

The experiment was carried out in a continuous way at a predetermined temperature of the bed by successively dosing at given time intervals three samples of each type of the silicone rubber composite. As it can be observed in the graphs illustrating the concentrations of gaseous components in time (Figure 3 and Figure 4), the concentration values within the same type of the composite demonstrate certain changeability. This is related to the dynamic character of the thermal decomposition process that was conducted in the fluidised bed. On the other hand, these differences are not significant in the majority of cases, and, after the conversion of the obtained values into mass contents, they permit to determine measurement uncertainties. 

The reading of the FTIR spectrum took place every 5 s, and, on its basis the concentrations of 18 gaseous components (that were selected, i.a. on the basis of previous research works [28]), were determined. The main gaseous products that are formed during thermal decomposition of silicone rubber composites, regardless of their composition and temperature, are CO_2_ and H_2_O. These are the final products of the oxidation of organic compounds contained in the polymer material, and, in spite of certain toxicity of carbon dioxide, their significant participation in the combustion products is beneficial from the environmental point of view. The contents of the remaining components, the majority of which exert a negative influence on living organisms and the environment, are much more important. In order to more easily illustrate the concentrations of these components, they were summed up in appropriate groups of compounds in Figure 3.

At a temperature of 500 °C (Figure 3a), the thermal decomposition of cross-linked silicone rubber (SR-1) resulted in the maximum instantaneous emission at the level of approximately 30 ppmv CO, 20 ppmv aldehydes, and approximately 15 ppmv methane. The instantaneous concentrations of cyclic siloxanes and NMHCs (non-methane hydrocarbons) did not exceed 5 ppmv. An addition of basalt flakes resulted in a decrease in instantaneous concentrations of CO and a simultaneous increase in instantaneous concentrations of cyclic siloxanes. This might indicate that the process of thermal decomposition (inhibition of the step of the decomposition of cyclic siloxanes) was slowed down by large surface particles. This is also confirmed by an increased time of the process of thermal decomposition of the samples SR-3. The sample containing basalt fibres (SR-3) demonstrated a different behaviour during thermal decomposition. Its decomposition time was approximately two times shorter than that of the reference sample, which led to the fact that the registered concentrations in flue gases of this composite were high (maximum approximately 40 ppmv CO, approx. 50 ppmv aldehydes, and approx. 5 ppmv cyclic siloxanes). This might indicate an indirect initiation of the processes of silicone rubber thermal decomposition by long basalt fibres. The fibres probably caused accelerated breaking and dividing the samples into smaller fragments, which resulted in a considerable increase in the surface of mass and heat transfer in the process.

It should be noted that the samples of the ceramizable composite, both the reference material (SR-4) and composites with an addition of basalt (SR-5, SR-6), emitted a similar amount of gaseous components (maximum approx. 10 ppmv aldehydes, approx. 6 ppmv methane, and approx. 6 ppmv CO). The introduction of the basalt filler resulted in an increased time of the thermal decomposition process and, consequently, decreased the instantaneous concentrations of, i.a. aldehydes. In this case, there was no differentiation between basalt flakes and fibres, since they only constituted a small part of additives. On the contrary to the basalt fibres in the sample SR-3, the basalt additives were successfully built into the polymer structure in SR-5 and SR-6, which was confirmed in the previous work.

An increase in the temperature of the thermal decomposition process to 600 °C (Figure 3b) resulted in a significant increase in its rate. This reduced the differences between particular composites, both in terms of the decomposition time and the concentrations of gaseous products resulting from their decomposition. The intensification of thermal decomposition contributed to a significant emission of the undesired compounds. This is the temperature of maximum instantaneous emission of aldehydes (approx. 150 ppmv for SR-1, SR-2, and SR-3), methane (approx. 100 ppmv for SR-1), as well as cyclic siloxanes (approx. 20 ppmv for composites without ceramizable additives). An addition of the basalt filler in the case of ceramizable composites resulted in a reduction of the instantaneous concentrations of CO, and also to a lesser extent of aldehydes and methane. The fact that the concentration of cyclic siloxanes does not increase may indicate accelerated decomposition of the samples SR-5 and SR-6 with reference to SR-4 as well as indirect catalysing of the oxidation process of CO to CO_2_ by basalt fillers.

At a temperature of 700 °C (Figure 3c), the majority of the formed organic components underwent oxidation, which caused the maximum emission of CO, at specific times exceeding 900 ppmv. The differences between particular composites almost completely disappeared. The only visible change could be a decreased emission of CO in the case of the composites containing ceramizable components with reference to the samples that did not contain such additives. By contrast, at a temperature of 800 °C (Figure 3d), an incomplete oxidation of CO to CO_2_ during the combustion of ceramizable composite samples occurred. Probably, at a temperature of 700 °C, the emission of CO in ceramizable composites also increased. However, the total emission of CO was lower than in the case of the composites without ceramizable additives, and the effect was unnoticeable, since the participation of the polymer matrix was smaller in these samples (a large number of additives).

The emission of nitrogen compounds, i.e. nitrogen oxides, ammonia, and hydrogen cyanide, is extremely important from the point of view of the environmental impact. In accordance with the expectations, the presence of these compounds was only detected in the case of ceramizable composites, since they contained a nitrogen source in the form of melamine cyanurate (MC). The obtained positive values of instantaneous concentrations of nitrogen compounds in Figure 4 in the case of composites not containing MC indicate typical fluctuations of the measurement apparatus (the algorithm conducting the deconvolution of FTIR spectra). 

At a temperature of 500 °C (Figure 4a), ammonia was the only nitrogen component that formed as a result of the decomposition of MC (the maximum instantaneous concentration approx. 10 ppmv). Additionally, at a temperature of 600 °C (Figure 4b), ammonia (the maximum instantaneous concentration approx. 28 ppmv) constituted the main nitrogen compound that formed during the thermal decomposition of ceramizable composites, but, at this temperature, it began to undergo oxidation to nitrogen oxides, mainly to N_2_O (the maximum instantaneous concentration approx. 17 ppmv). At a temperature of 700 °C (Figure 4c), the additive MC contributed to the emissions of all the above-mentioned nitrogen compounds at instantaneous concentrations exceeding maximum approx. 20 ppmv. A particularly high instantaneous concentration was obtained in the case of N_2_O (maximum approx. 80 ppmv), which, also at a higher temperature, 800 °C (Figure 4d), constituted the dominant nitrogen component (maximum approx. 110 ppmv). Under such conditions, ammonia underwent almost complete oxidation, but mainly to N_2_O, since the residence time of gases turned out to be insufficient for the further oxidation of nitrogen. The impact of the basalt filler on a type and amount of the emitted nitrogen compounds was negligible, regardless of the temperature of the thermal decomposition process.

It is interesting to notice that, during the combustion of the ceramizable composites samples at a temperature of 800 °C, there is no effective oxidation of CO to CO_2_ and N_2_O to NO. It is possible that the temperature or the process conditions (a good heat exchange in fluidised bed) are sufficient for allowing the process of ceramization in the bed. The formed ceramic layer effectively reduces the diffusion of oxygen to the reaction zone, whereas its isolating character limits the processes of thermal decomposition. Such an effect might also be due to the presence of the MC, whose degradation proceeds gradually and the time that is necessary for complete oxidation of this compound turned out to be too long in relation to the residence time of gases in the fluidised bed reactor.

### 3.3. Balance of Thermal Degradation Process

The masses of the obtained components were calculated on the basis of the obtained graphs illustrating the concentrations of gaseous components during the process of thermal decomposition of silicone rubber composites. In this way, an amount of the formed gaseous components expressed as the sample mass was determined (Table 2), the composition of the formed gases in mass percentages was presented (Table 3) and the balance of carbon atoms was calculated (Table 4).

The addition of the basalt filler to the samples of composites not containing ceramizable additives resulted in a decreased emission of gases at all of the analysed temperatures (Table 2). No clear difference in the amounts of the formed gases between the composites containing basalt flakes and fibres was noticed. Different dependencies were obtained in the case of ceramizable composites. At a temperature of 500 °C, the emission of gases during thermal decomposition was similar for all of the composites, whereas at 600 °C the addition of the basalt filler had a negative impact on the amount of the formed gases. The sample (SR-5) with the addition of basalt flakes demonstrated a particularly increased emission at this temperature. Additionally, at higher temperatures, the introduction of basalt flakes turned out to be less beneficial in relation to the presence of basalt fibres. On the other hand, only the analysis of the composition of the gaseous products made it possible to assess the impact of the basalt filler on the level of toxic emissions of gaseous components.

Having converted the data, it turned out that the sample containing basalt fibres, whose decomposition took place very dynamically at 500 °C (SR-3) emitted gases of a similar composition in comparison to the reference material (Table 3). The only difference consisted in increased participation of aldehydes and decreased participation of hydrocarbons. However, the sample containing basalt flakes emitted gaseous products with increased participation of cyclic siloxanes in exchange for lower participation of CO in comparison to SR-1. This proves a change of the mechanism of the thermal decomposition of samples containing these fillers. The basalt fibre intensifies the whole decomposition process, which increases the contact surface and facilitating the oxidation processes. Basalt flakes, in turn, inhibit the decomposition process of cyclic siloxanes through an opposite phenomenon, by limiting the access of air to the sample. 

The same dependencies took place at a temperature of 600 °C, although apparently the addition of both basalt fillers contributed in the same way to slowing down the process of thermal decomposition of gaseous components (increased participation of cyclic siloxanes and aldehydes in relation to SR-1). The addition of basalt flakes (SR-2) acted as a reducing diffusion factor, due to which the decomposition and oxidation process was stopped. However, basalt fibres probably, similarly like at a lower temperature, caused the violent decomposition of the sample. In consequence, the resulting gases did not manage to undergo further degradation and oxidation, which is proven by a lower participation of CO_2_ in exchange for CO in flue gases originating from the decomposition of SR-3. At higher temperatures, the kinetics of the degradation and oxidation processes resulted in the absence of significant differences between the samples without ceramizable additives.

The impact of the addition of basalt fillers in ceramizable composites on the quality of the formed gases was ambiguous. At temperatures of 500 °C and 600 °C, both additives (in particular basalt flakes) contributed to an improvement in the oxidation process. This was demonstrated by the high participation of CO_2_ and low participation of CO, aldehydes, and cyclic siloxanes at the same time. On the other hand, at higher temperatures in the samples containing basalt fillers, the oxidation of CO to CO_2_ was limited. Both of these phenomena (except for the above-mentioned in Section 3.2) may be explained by the accelerated fragmentation of the sample (a similar effect like for SR-3 at 500 °C), which intensifies the oxidation process at lower temperatures but does not permit the gases to be present for a sufficiently long time at higher temperatures.

The carbon balance was calculated by dividing the mass of carbon atoms in the gaseous components formed in the process of thermal decomposition by %C in the composite sample on the basis of a theoretical mass of carbon in the samples (%C in Table 1) (Table 4). At a temperature of 800 °C the obtained balance was close to 100% (taking into account the measurement uncertainty). This leads to the conclusion that at this temperature all the organic compounds were burnt out and entered the gas phase. Moreover, this also proves that the conducted analysis and compilation of the results from the FTIR analyser were correct. At lower temperatures the carbon balance amounted, on average, to 87%, 63%, and 48%, at a temperature of 700 °C, 600 °C, and 500 °C, respectively. This is caused by the formation of the carbon deposit (char) which is thermally stable and, in spite of the conditions in the bed, does not undergo burnout and remains in the form of ash. After each of the experiments at a given temperature, the bed was heated to a temperature higher than 800 °C in order to confirm the presence of carbon in the ash resulting from the combustion of the samples at temperatures lower than 800 °C. In accordance with the expectations, during the heating of the bed the emission of carbon compounds (HCHO, CO, and CO_2_) was observed.

### 3.4. Toxicity Evaluation

The mean toxicometric index was used in order to evaluate the toxicity of the gases resulting from the process of thermal decomposition of rubber silicone composites. This is the sum of the toxicometric indices of particular gaseous components (Equation (1)). The toxicometric index is defined as the normalized volume (in m^3^) of toxic gases emitted from the combustion of 1 mg of the sample (Equation (2)). Hence, the higher the value of the index, the higher toxic a given material is. The normalized concentration values that were customarily assumed while calculating the values of the toxicometric index are the LCs (lethal concentrations) values [9,17,18,29]. However, it was decided to substitute the LCs values by introducing more universal values—Threshold Limit Values—in the order of eight-hour time weighted averages (TLV®-TWA) due to the lack of experimental data giving the LCs values for all the components analysed in the present work. TLV® is a level of a toxic substance concentration to which people can be exposed without adverse effects and it was established by the American Conference of Governmental Industrial Hygienists (ACGIH®) [30].

Therefore, toxicometric index *W_mean_* in this case can be calculated according to the equations:

(1) $$W_{mean}=\overset{n}{\sum }W$$

where *n* is a number of toxic gases.

(2) $$W=\frac{E}{TLV-TWA}$$

where *E* is a specific emission of a given compound, derived from FTIR analysis, g/g.

The proposed method enables including all emitted gases in toxicity evaluation without the need of additional unnecessary animal testing. Although it does not provide a direct value stating whether a given material is toxic, it can be easily adopted to compare toxic hazard in the case of fire for different materials emitting multicomponent gas mixtures.

**Figure 5:**
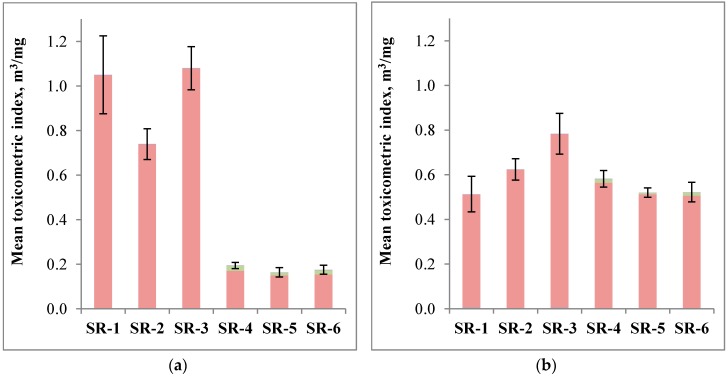

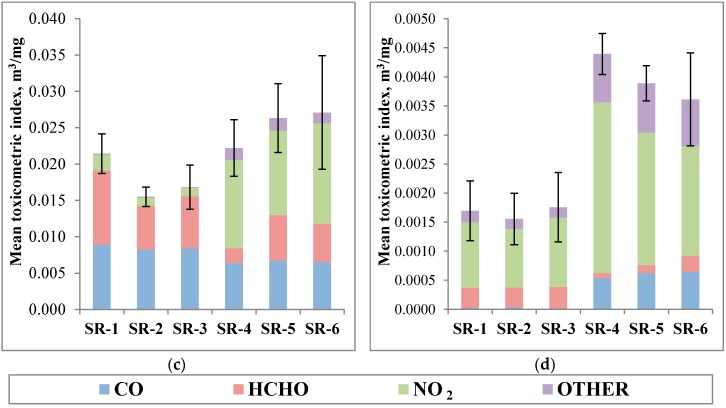
Toxicometric indices evaluated for silicon rubber composites: (**a**) at 500 °C; (**b**) at 600 °C; (**c**) at 700 °C; and, (**d**) at 800 °C. (Note: different scales in each graph).

Figure 5 illustrates the determined values of toxicometric indices of the analysed composites, at temperatures of 500, 600, 700, and 800 °C, respectively. At T = 500 °C, the values of toxicometric indices of ceramizable composites are decisively lower than the values of indices of silicone rubber composites (SR-1, SR-2, and SR-3) due to the presence of formaldehyde. This indicates a decisively lower toxicity of the gaseous products of thermal decomposition, which are formed as a result of the decomposition of ceramizable composites in relation to the samples SR-1, SR-2, and SR-3. It should also be clearly highlighted that the addition of the basalt filler in the form of BFL reduces the toxicity of the decomposition products of composite SR-2 in relation to the reference composite SR-1 (Figure 5a).

At a temperature of 600 °C, both ceramizable composites and the composites only containing the basalt filler are characterized by a higher toxicity of gaseous products of thermal decomposition in relation to the reference sample. This is caused by the emission of a higher amount of formaldehyde, which might probably be a result of inhibiting the oxidation processes, resulting from the above-described effect of the basalt filler. A slightly higher toxicity of ceramizable composites in relation to SR-1 additionally results from the emission of NO_2_, which is a product of the oxidation of nitrogen compounds resulting from the MC decomposition (Figure 5b).

Within the temperature range T = 700 °C, the toxicometric index decreased its value by an order of magnitude, which translates into a significant reduction of toxicity of the emitted gases. At a temperature of 700 °C (Figure 5c), the value of the toxicometric index was mainly affected by CO in the case of the samples SR-2 and SR-3 and NO_2_ in the case of the samples of ceramizable composites. A further significant reduction of toxicity of gaseous products of the thermal decomposition and combustion of the analysed composites takes place at T = 800 °C. However, it should be noted that, at this temperature, ceramizable composites emit decisively more toxic gases than the composites only containing the basalt filler. An increase in the toxicity of the gaseous decomposition products of ceramizable samples is related to the presence of a considerable amount of NO_2_ (Figure 5d).

## 4. Conclusions

At a temperature below 700 °C, the mechanism of the process of thermal decomposition undergoes a modification for the samples containing basalt fillers in relation to the reference sample. A microscopically heterogeneous structure of the composite that results from the introduction of basalt fibres into the cross-linked silicone rubber (sample SR-3) intensifies the process of its physical fragmentation. The sample is divided into smaller fragments at the interface between basalt and the remaining part of the material, which considerably increases the surface of mass and heat exchange in the process. Consequently, the process of the thermal decomposition and emission of gaseous components is accelerated. As a result, at both 500 °C and at 600 °C, the accelerated decomposition of the sample limited the possibility of effective oxidation of the resulting gases. On the other hand, the addition of basalt flakes to the silicone rubber (sample SR-2) creates a thermal barrier and limits the access of air to the sample, thus inhibiting the process of the thermal decomposition of the material. At a temperature lower than 700 °C, the gases formed as a result of the thermal decomposition of composites are particularly toxic, owing to the presence of significant concentrations (up to 150 ppmv) of formaldehyde. It does not undergo oxidation to more stable inorganic products, due to the insufficient availability of radicals in the gaseous phase, which are necessary for its oxidation. 

At a temperature of 600 °C, the toxicity of gases was similar for the samples with ceramizable additives ($W_{mean}$: 0.5–0.6) and the ones without these additives ($W_{mean}$: 0.5–0.8). In the former case, a beneficial synergistic effect of the addition of basalt flakes or fibres with mineral additives ($W_{mean}$ decreased by approx. 10% for the samples SR-5 and SR-6 in relation to SR-4) could be observed. Surprisingly, the basalt fillers themselves increased the toxicity of gases, which has been explained above. However, at this temperature, ceramizable additives did not play a crucial role in reducing the toxicity of gases (similar, best values $W_{mean}$ for the samples SR-1 and SR-5, SR-6). The situation is different at a temperature of 500 °C, at which ceramizable additives reduce the toxicity of gases between five and six times. Afterwards, a synergy with basalt additives (an additional decrease in the value $W_{mean}$ by 16% and 10%) may be observed.

At higher temperatures of polymer decomposition, the exponentially growing rate of the oxidation reaction in the gaseous phase leads to the lack of significant differences between the toxicity of gases for the samples with and without basalt additives. The value of the toxicometric index at a temperature of 700 °C and 800 °C was one or two orders of magnitude lower than the one that was observed within the temperature range of 500–600 °C. This indicates an emission of considerably less toxic gases, since inorganic components, such as H_2_O, CO_2_, CO, and NO_x_, appear in place of formaldehyde.

## Figures and Tables

**Table 1:** Composition (in phr—parts per hundred parts of rubber) of the composite mixes.

Composite type	SR-1	SR-2	SR-3	SR-4	SR-5	SR-6
SR	100	100	100	100	100	100
DCP	2.5	2.5	2.5	2.5	2.5	2.5
Silica	-	-	-	15	15	15
MC	-	-	-	30	30	30
CaCO_3_	-	-	-	15	15	15
Glass frit	-	-	-	30	30	30
ZnB	-	-	-	15	15	15
BFL	-	20	-	-	20	-
BFS	-	-	20	-	-	20
%C	34	28	28	22	20	20

DCP—dicumyl peroxide; MC—melamine cyanurate; CaCO3—calcium carbonate; ZnB—zinc borate; BFL—basalt flakes; BFS—basalt fibres; %C—carbon content in the sample. Silicone rubber composites: SR-1, SR-2, SR-3; Ceramizable silicone rubber composites: SR-4, SR-5, SR-6.

**Table 2:** Sum of masses of gaseous products emitted from the combustion of composites, g/g of sample.

Temperature	500 °C	600 °C	700 °C	800 °C
SR-1	0.44 ± 0.02	0.57 ± 0.03	1.44 ± 0.03	1.81 ± 0.04
SR-2	0.33 ± 0.02	0.47 ± 0.03	1.20 ± 0.09	1.52 ± 0.07
SR-3	0.34 ± 0.02	0.48 ± 0.02	1.18 ± 0.03	1.47 ± 0.10
SR-4	0.32 ± 0.02	0.35 ± 0.02	0.76 ± 0.06	1.11 ± 0.08
SR-5	0.31 ± 0.01	0.43 ± 0.01	0.73 ± 0.02	1.06 ± 0.05
SR-6	0.33 ± 0.01	0.38 ± 0.01	0.61 ± 0.01	0.98 ± 0.07

**Table 3:** Composition of gases emitted from silicone rubber composites combustion, wt.%.

	H_2_O	CO_2_	CO	NOx	CH_4_	NMHCs *	Aldehydes	Siloxanes	NH_3_+HCN
**500 °C**									
SR-1	0.0 ± 0.0	0.0 ± 0.0	16.7 ± 5.4	0.9 ± 1.0	9.6 ± 5.2	2.4 ± 2.0	29.5 ± 4.1	39.0 ± 11.3	1.8 ± 0.3
SR-2	0.0 ± 0.0	0.0 ± 0.0	6.0 ± 1.9	1.1 ± 1.2	7.1 ± 1.8	2.3 ± 1.1	29.7 ± 5.8	52.3 ± 6.3	1.7 ± 0.2
SR-3	0.0 ± 0.0	0.0 ± 0.0	15.1 ± 1.7	0.9 ± 0.7	4.3 ± 0.9	1.1 ± 1.2	40.5 ± 6.5	37.2 ± 6.6	1.5 ± 0.9
SR-4	0.0 ± 0.0	57.7 ± 2.4	2.3 ± 0.4	5.5 ± 1.6	0.9 ± 0.5	1.2 ± 1.0	7.2 ± 1.3	21.4 ± 2.9	1.2 ± 0.5
SR-5	0.0 ± 0.0	61.5 ± 2.6	2.5 ± 0.3	4.3 ± 1.0	2.7 ± 2.5	1.4 ± 1.4	6.6 ± 1.5	16.8 ± 3.4	1.4 ± 0.7
SR-6	0.0 ± 0.0	60.6 ± 6.1	2.0 ± 0.7	5.1 ± 0.8	1.6 ± 0.9	2.0 ± 1.6	7.1 ± 2.1	18.0 ± 5.5	1.2 ± 0.4
**600 °C**									
SR-1	0.0 ± 0.0	37.8 ± 4.3	28.4 ± 4.6	0.1 ± 0.1	5.6 ± 3.1	1.0 ± 0.8	11.7 ± 1.2	15.2 ± 5.9	0.3 ± 0.2
SR-2	0.0 ± 0.0	33.7 ± 3.0	26.1 ± 4.6	0.1 ± 0.1	3.4 ± 0.7	0.8 ± 0.6	17.0 ± 1.1	18.4 ± 2.3	0.4 ± 0.2
SR-3	0.0 ± 0.0	18.0 ± 2.0	36.0 ± 3.4	0.2 ± 0.1	3.1 ± 0.2	0.9 ± 0.8	20.8 ± 2.7	20.4 ± 3.5	0.6 ± 0.2
SR-4	0.0 ± 0.0	23.2 ± 1.5	25.5 ± 3.4	5.9 ± 1.3	2.0 ± 0.2	1.0 ± 0.7	20.8 ± 1.7	17.6 ± 2.4	4.1 ± 1.2
SR-5	0.0 ± 0.0	47.9 ± 0.9	11.2 ± 3.7	3.8 ± 0.9	1.5 ± 0.4	0.6 ± 0.7	15.2 ± 1.3	17.0 ± 4.0	2.8 ± 0.6
SR-6	0.0 ± 0.0	44.6 ± 1.9	11.4 ± 3.4	4.5 ± 1.0	1.6 ± 0.4	0.8 ± 0.6	16.6 ± 1.7	17.6 ± 3.9	2.8 ± 0.2
**700 °C**									
SR-1	35.8 ± 3.1	46.0 ± 2.1	17.7 ± 1.8	0.1 ± 0.0	0.3 ± 0.1	0.1 ± 0.1	0.1 ± 0.0	0.0 ± 0.0	0.1 ± 0.1
SR-2	36.1 ± 1.6	43.5 ± 1.6	19.8 ± 1.7	0.0 ± 0.0	0.3 ± 0.1	0.1 ± 0.0	0.1 ± 0.1	0.0 ± 0.0	0.1 ± 0.1
SR-3	39.5 ± 1.9	39.5 ± 5.3	20.4 ± 3.4	0.1 ± 0.1	0.3 ± 0.0	0.1 ± 0.0	0.1 ± 0.1	0.0 ± 0.1	0.0 ± 0.0
SR-4	18.6 ± 2.3	50.2 ± 1.6	24.0 ± 3.2	4.7 ± 0.6	0.3 ± 0.1	0.2 ± 0.2	0.1 ± 0.1	0.0 ± 0.0	2.0 ± 0.3
SR-5	23.4 ± 2.7	42.2 ± 2.2	26.3 ± 1.1	5.2 ± 0.6	0.3 ± 0.1	0.2 ± 0.2	0.4 ± 0.2	0.0 ± 0.1	2.0 ± 0.3
SR-6	14.6 ± 2.2	46.3 ± 4.4	30.2 ± 1.6	5.7 ± 0.8	0.4 ± 0.0	0.4 ± 0.2	0.3 ± 0.1	0.1 ± 0.1	2.0 ± 0.4
**800 °C**									
SR-1	32.3 ± 3.4	67.5 ± 3.3	0.0 ± 0.0	0.0 ± 0.0	0.0 ± 0.0	0.0 ± 0.0	0.0 ± 0.1	0.0 ± 0.0	0.0 ± 0.0
SR-2	32.8 ± 1.2	67.0 ± 1.2	0.1 ± 0.0	0.0 ± 0.0	0.0 ± 0.0	0.0 ± 0.0	0.0 ± 0.1	0.0 ± 0.0	0.0 ± 0.0
SR-3	28.2 ± 0.9	71.6 ± 0.8	0.0 ± 0.0	0.0 ± 0.0	0.0 ± 0.0	0.0 ± 0.0	0.1 ± 0.1	0.0 ± 0.0	0.0 ± 0.0
SR-4	24.1 ± 2.9	71.4 ± 1.9	1.4 ± 0.8	2.6 ± 0.3	0.0 ± 0.0	0.0 ± 0.1	0.1 ± 0.0	0.0 ± 0.0	0.4 ± 0.1
SR-5	27.8 ± 1.4	67.0 ± 1.3	1.7 ± 0.2	2.9 ± 0.5	0.0 ± 0.0	0.0 ± 0.1	0.1 ± 0.2	0.0 ± 0.0	0.4 ± 0.0
SR-6	21.9 ± 1.5	72.8 ± 2.2	1.9 ± 0.7	2.8 ± 0.4	0.0 ± 0.0	0.0 ± 0.0	0.1 ± 0.1	0.0 ± 0.0	0.4 ± 0.1

NMHCs—non-methane hydrocarbons.

**Table 4:** Carbon balance (out/in) resulting from combustion of composites, %.

Temperature	500 °C	600 °C	700 °C	800 °C
SR-1	55.1 ± 1.4	62.8 ± 4.1	88.0 ± 6.3	100.0 ± 5.4
SR-2	46.9 ± 2.7	61.3 ± 4.6	88.2 ± 3.9	99.7 ± 3.2
SR-3	47.2 ± 1.3	66.8 ± 3.2	83.3 ± 1.8	102.6 ± 5.9
SR-4	43.2 ± 3.1	54.7 ± 4.1	87.6 ± 8.6	105.2 ± 6.4
SR-5	46.6 ± 1.5	68.4 ± 1.4	89.1 ± 1.7	103.9 ± 5.8
SR-6	49.7 ± 1.5	61.8 ± 2.3	83.4 ± 1.9	104.9 ± 5.8
